# Cardiac Function in Newborns with Congenital Hypothyroidism: Association with Thyroid-Stimulating Hormone Levels

**DOI:** 10.4274/jcrpe.1969

**Published:** 2015-12-03

**Authors:** Taliha Öner, Rahmi Özdemir, Önder Doksöz, Yılmaz Yozgat, Cem Karadeniz, Savaş Demirpençe, Murat Muhtar Yılmazer, Muammer Büyükinan, Timur Meşe, Vedide Tavlı

**Affiliations:** 1 Dr. Behçet Uz Children’s Hospital, Clinic of Pediatric Cardiology, İzmir, Turkey; 2 Konya Training and Research Hospital, Clinic of Pediatric Endocrinology, Konya, Turkey

**Keywords:** newborn, congenital hypothyroidism, cardiac functions

## Abstract

**Objective:**

The aims of this study were to demonstrate ventricular function changes in patients with congenital hypothyroidism and to investigate whether there is an association between any such changes and thyroid-stimulating hormone (TSH) levels using M-mode and Doppler electrocardiography (ECG) and tissue Doppler imaging (TDI).

**Methods:**

Twenty-seven patients 5-30 days of age with congenital hypothyroidism who were scheduled to receive L-thyroxine treatment and 20 healthy newborns were included in this study. Twelve-lead ECG and M-mode TDI recordings of the patient and healthy groups were obtained. The patient group was divided into two subgroups according to TSH level (>100 uIU/mL or <100 uIU/mL), which were then compared on all parameters.

**Results:**

Decreases were observed in the ejection fraction (EF), shortening fraction (SF), and mitral lateral annulus, mitral septal annulus, and tricuspid lateral annulus systolic velocity (Sa) on TDI, whereas left ventricular end-systolic diameter (LVESd) and corrected QT interval (QTc) dispersion were significantly increased in the patient group compared with the control group. No significant differences between the groups were found in left ventricular end-diastolic diameter (LVEDd) or heart rate. When the two patient subgroups (TSH >100 uIU/mL and <100 uIU/mL) were compared, TDI septal annulus Sa wave length and heart rate were significantly lower in the TSH >100 group.

**Conclusion:**

Impairment in left ventricular systolic function and increased risk of arrhythmia were observed in newborn infants with congenital hypothyroidism. TSH level was associated with heart rate and interventricular septum velocity.

**WHAT IS ALREADY KNOWN ON THIS TOPIC?**

Impaired left ventricular contraction and relaxation are observed in adults with hypothyroidism.

**WHAT THIS STUDY ADDS?**

Our results showed that congenital hypothyroidism can reduce the left ventricular systolic function and increase the risk of arrhythmia in newborn. Thyroid-stimulating hormone level is associated with heart rate and interventricular septum velocity.

## INTRODUCTION

The incidence of congenital hypothyroidism in newborns varies between 1/3,500 and 1/4,000. As a result of the recent introduction of screening programs for congenital hypothyroidism also in Turkey, early diagnosis is now possible, and the number of children with cognitive delay due to hypothyroidism has decreased. Primary hypothyroidism is characterized by an increased level of blood thyroid-stimulating hormone (TSH) and decrease in free triiodothyronine (fT3) and free thyroxine (fT4) levels (1).

The effects of thyroid hormones on the cardiovascular system have been well documented. Cardiac findings in hypothyroidism include pericardial effusion, weak arterial pulse, bradycardia, hypotension, facial and peripheral edema, deepened cardiac sounds, and congestive heart failure findings such as ascites, orthopnea, and paroxysmal dyspnea (2,3). Chronotropic response and normal tension of the heart muscle during diastole are due to T3. Moreover, triiodothyronine affects the number of B adrenergic receptors and their sensitivity to catecholamines (2). Cardiogenic shock, when present in a patient with hypothyroidism, demonstrates poor response to catecholamines. Severe hypothyroidism results in muscle and isovolemic relaxation, increased contraction times, and an increase in the myocardial performance index of systolic and diastolic function (4). Sinus bradycardia, low QRS voltage, prolonged QT interval, low P-wave amplitude, right branch blockage, ventricular dysrhythmia, and torsades de pointes may be seen on electrocardiography (ECG) in hypothyroidism (2,3,4,5). These cardiovascular effects can be reversed by T4 treatment.

The aim of this study was to determine whether cardiac function was affected in newborns with hypothyroidism and to identify the association of any such effect with TSH levels.

## METHODS

Twenty-seven patients aged 5-30 days who were brought to the Dr. Behçet Uz Children’s Diseases and Surgery Education and Research Hospital in İzmir, Turkey, between October 2009 and May 2010 with high TSH levels and who were scheduled to receive treatment were included in the study. Twenty age- and gender-matched healthy newborns with normal physical and cardiologic examination results served as controls. The patient group was divided into two subgroups: those with TSH levels <100 uIU/mL and those with TSH >100 uIU/mL.

### Electrocardiography:

Twelve-lead ECG recordings were obtained in all patients. The QT interval was taken as the period from the beginning of the Q wave to the end of the T wave, defined as the point at which the T wave converted to the TP isoelectric line. When a U wave was present, the end of the T wave was defined as the lowest point between the T and U waves. QT dispersion was defined as the difference between the maximal and minimal QT intervals on 12-channel standard ECG. The QT intervals on ECGs were corrected using Bazett’s formula [corrected QT interval (QTc)=QT/√R-R] and were expressed as QTc (6).

### Echocardiography:

Transthoracic two-dimensional Doppler ECG was performed using GE Vivid 3 equipment (GE Healthcare, Milwaukee, WI) and a 7S transducer. M-mode echocardiography measurements were obtained at the level of the posterior mitral valve, per the recommendations of the American Echocardiography Society (7).

### Tissue Doppler imaging (TDI):

Pulsed wave (PW) Doppler sampling volume and Nyquist limit in the TDI program using a 7 MHz probe were adjusted to 2-4 mm and 15-20 cm/s, respectively, and the gain was adjusted to receive minimal noise and thus provide clear tissue signals. In the apical four-chamber image, the sample volume was placed at the junction of the left ventricular (LV) free wall, the mitral valve annulus, the mitral septal wall, the right ventricular (RV) free wall, and the tricuspid annulus. The recording was performed over at least five cardiac cycles, during calm periods, so that the flow was not affected by respiration. Systolic velocity (Sa) waves were recorded from the mitral lateral, mitral septal, and tricuspid lateral walls (8).

### Statistical Analysis

Statistical analysis was performed using the SPSS for Windows software package (ver. 17; SPSS; Chicago, IL, USA). The Kolmogorov-Smirnov test was used to investigate the normality of distribution of the variables. Student’s t-test and the Mann-Whitney U-test were used for variables with and without normal distribution, respectively, to detect any differences between the control and patient groups. A value of p<0.05 was taken to indicate statistical significance.

## RESULTS

Decreases were observed in the ejection fraction (EF), shortening fraction (SF), and mitral lateral annulus, mitral septal annulus, and tricuspid lateral annulus Sa on TDI (patient group: 68.56±6.04, 35.96±4.59, 5.12±0.81, 3.85±0.61, and 7.64±1.41, respectively; control group: 72.63±4.77, 39.00±3.75, 5.83±1.24, 4.89±0.99, and 9.31±1.77, respectively), whereas left ventricular end-systolic diameter (LVESd) and QTc dispersion were significantly increased in the patient group compared with the control group (patient group: 1.16±0.13 and 0.068±0.030, respectively; control group: 1.04±0.20 and 0.036±0.014, respectively). No significant differences were detected in left ventricular end-diastolic diameter (LVEDd) or heart rate between the two groups (Table 1).

When the two patient subgroups (TSH <100 uIU/mL and >100 uIU/mL) were compared, the TDI septal annulus Sa wave velocity and heart rate were significantly decreased in the TSH >100 compared with the TSH <100 group (3.55±0.52, 148±16; vs. 4.07±0.59, 162±11, respectively) (Table 2).

## DISCUSSION

Impaired LV contraction and relaxation are observed in adults with hypothyroidism, commensurate with their decreased thyroid hormone levels; this condition is reversible with treatment (9,10,11,12,13). The current study revealed that even during the newborn period, the systolic functions of the heart are affected, the tendency toward arrhythmia is increased due to increased QTc dispersion, and TSH levels are associated with heart rate and interventricular septal velocity in hypothyroidism. When assessing LV diastolic performance, TDI is more reliable than conventional Doppler, because it is not as affected by loading conditions; furthermore, TDI measurements are more reproducible (14). Diastolic abnormalities can be detected using TDI before the onset of hemodynamic abnormalities can be identified on conventional echocardiography (15). Therefore, TDI is superior to and more sensitive than conventional echocardiography for the detection of subclinical abnormalities in the heart. TDI can be used to evaluate global and regional ventricular performance in a variety of clinical conditions. Pulsed TDI of the tricuspid annulus represents a non-invasive and reliable method of assessing RV function compared with the gold standard of magnetic resonance imaging (16). Because diastolic dysfunction starts before systolic dysfunction, tissue Doppler echocardiography may be helpful for early diagnosis of diastolic dysfunction (17,18). Harada et al (19) suggested that an insufficient increase in Sa indicates an impaired response of the right ventricle to exercise in patients with tetralogy of Fallot. The Sa value obtained from the myocardial segments can be used to evaluate segmental systolic function. Regional systolic functional impairment can be defined by TDI in ischemic heart disease. A decrease in Sa rate was reported in infarct areas (20), but Sa values were decreased compared with controls also in ischemic segments without infarct. Sa values were also decreased in patients with dilated cardiomyopathy, hypertrophic cardiomyopathy, valvular heart disease, or hypertensive heart diseases. In a study of 50 neonates with congenital hypothyroidism and 35 control neonates performed by Mao et al (21) right and LV Sa values were low. Similarly, in the present study, the Sa wave velocity of the mitral lateral, mitral septal, and tricuspid lateral annuli were decreased in the group with hypothyroidism compared with the control group.

Despite recent developments resulting from improved understanding of myocardial mechanics, clinical measurement of EF, LV systolic volume change, SF, and the percentage change in systolic diameter represent the most commonly used methods of assessing ventricular function (22). In a study of 40 patients with congenital hypothyroidism and 30 normal controls, Mao et al (23) found that LV EF was decreased in the hyperthyroid group compared with the controls. Similarly, in the present study, EF and SF were decreased in the group with hypothyroidism compared with the control group.

Increased QT interval dispersion (QTd) is an electrocardiographic parameter that has been shown to be associated with malignant ventricular arrhythmias and sudden death, and QTc for heart rate has emerged as a potentially important predictor of cardiac death. Increased QTd is directly related to TSH levels in overt hypothyroidism and mean QTc was reported to be significantly increased in subclinical hypothyroidism compared with controls in several studies (24). Similarly, in the current study, QTc dispersion was increased significantly in the patient group compared with controls. However, no association was detected between TSH levels and QTc dispersion.

Sympatho-vagal regulation of heart rate is diminished in hypothyroidism. QTc dispersion is decreased significantly following treatment; however, PR and RR intervals and the duration of QRS remain unchanged (25). In a study performed on 12 patients with primary hypothyroidism using PW echocardiography, LV posterior wall and septal thickness were increased in patients with hypothyroidism, and heart rate was also reported to be increased 6 months after treatment (26). In the current study, TSH levels were associated with heart rate and interventricular septum velocity.

We are aware that our study had some limitations. In this context, difficulties in recording tissue Doppler flow rates due to tachycardia in newborn patients need to be mentioned. The study would have benefited from a larger number of subjects, and follow-up information on the patients would also have improved the study.

In conclusion, congenital hypothyroidism during the neonatal period causes impairment in the systolic functions of the heart and increases QT dispersion. Heart rate and the interventricular septal wall are affected by increased TSH levels.

## Figures and Tables

**Table 1:**
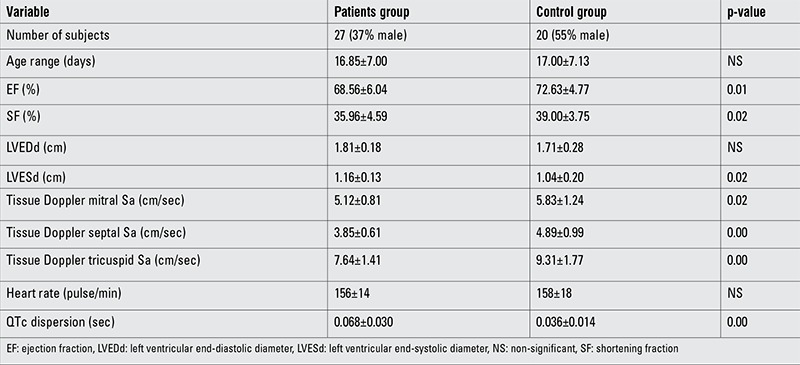
Comparison of the data of the total group of patients and control group (mean ± standard deviation)

**Table 2:**
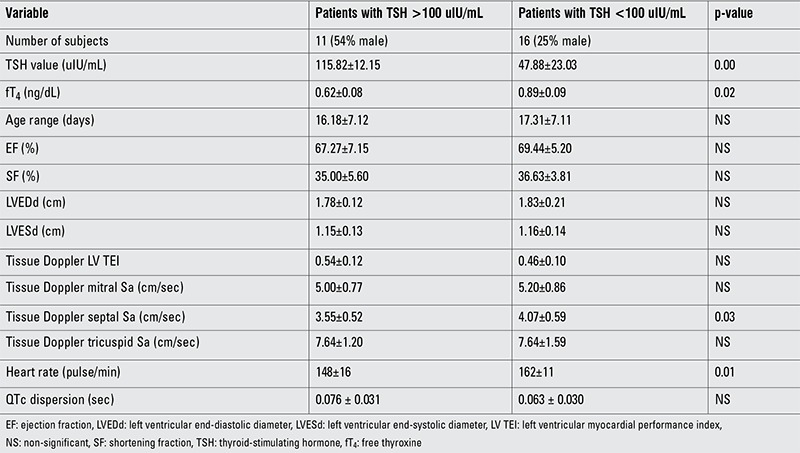
Comparison of the data (mean ± SD) of the patient subgroups with thyroid-stimulating hormone greater than and less than 100 uIU/mL
